# Diagnostic Accuracy of Sixty Four Multi-Slice CT Angiography in Assessment of Arterial Cut-Off and Run-Off in Comparison with Surgical Findings

**Published:** 2011-09-25

**Authors:** M. Noaparast, A. Rabani, F. Karimian, M. Bodaghabadi, S. Aran, R. Mirsharifi, A. Jafarian, F. Vaezi, H. Ghanaati

**Affiliations:** 1 Assistant Professor, Department of Surgery, Shohadaye Ashayer Hospital, Lorestan University of Medical Sciences, Khorramabad, Iran; 2 Full Professor, Department of Surgery, Imam Khomeini Hospital, Tehran University of Medical Sciences, Tehran, Iran; 3 Assistant Professor, Department of Surgery, Imam Khomeini Hospital, Tehran University of Medical Sciences, Tehran, Iran; 4 General Physician, Iranian Center for Breast Cancer (ICBC), Tehran University of Medical Sciences, Tehran, Iran; 5 General Physician, Massachusetts General Hospital, Harvard Medical School, Boston, MA, USA; 6 Associate Professor, Department of Surgery, Imam Khomeini Hospital, Tehran University of Medical Sciences, Tehran, Iran; 7 Medical Student, Tehran University of Medical Sciences, Tehran, Iran; 8 Associate Professor, Department of Radiology, Advanced Diagnostic and Interventional Radiology Research Center(ADIR), Medical Imaging Center, Imam Khomeini Hospital, Tehran University of Medical Sciences, Tehran, Iran

**Keywords:** Sixty Four Multi-Slice CT Angiography, Surgery, Arterial Disease, Cut-Off, Run-Off Site, Diagnostic Accuracy

## Abstract

**Background/Objective:**

The accurate anatomic mapping and determination of the severity of arterial disease, an important health problem of the elderly, is of great significance. We aimed to determine the diagnostic value of 64-multislice CT angiography (MSCTA) in run-off and cut-off sites of arterial disease.

**Patients and Methods:**

Throughout the study, MSCTA followed by an operative intervention was carried out on a total of 38 patients with clinical signs and symptoms suggestive of arterial disease (AD) all of whom had the indication for vascular surgery. The mean age of patients was 34±15.86 (range, 23 to 93) years. MSCTA was executed using a 64-slice CT scanner, during the arterial phase of injecting the nonionic, contrast medium with a power injector at the rate of 5 ml/sec into the antecubital vein and exploration and revascularization of peripheral arterial disease was performed intraoperatively.

**Results:**

Atherosclerosis and arterial disease, the most common causes of vascular occlusion, were more common in the lower extremities. According to MSCTA findings, the most frequent site of stenosis was the superficial femoral artery. Spearman’s correlation coefficient showed a high degree of agreement amongst the raters. The sensitivity, specificity, positive predictive value (PPV), negative predictive value (NPV) and the accuracy of MSCTA compared to surgery were 83.8%, 96%, 96.8%, 81.3% and 89%, respectively. MSCTA findings were compared with surgery as a standard of reference, which showed concordance in the majority of cases (81.6%). Cut-off sites were correctly identified by MSCTA in 97.3% of the patients and the most common sites of discordance were the run-off sites (18.2%).

**Conclusion:**

MSCTA angiography as a novel diagnostic modality may be a suitable alternative and a viable choice for routine clinical diagnosis.

## Introduction

Arterial disease (AD) is a consequential health problem of developed countries that has influence on a large segment of the adult population with an age adjusted prevalence of 4% to 15% affecting more than 5 million adults in the United States.[1][2] Peripheral vascular atherosclerosis is the main etiology of intermittent claudication and cramping in the population older than 60 years. Surgical revascularization is the preferred strategy for patients who develop critical ischemia. Determination of the severity of stenosis and anatomic mapping of vasculature is fundamental for the diagnosis of the occlusive sites.[3] Moreover, the accurate localization and determination of the severity of arterial involvement is of great significance.[4]

Although conventional angiography is the gold standard and diagnostic modality of choice for AD, it is invasive, expensive and associated with morbidity and mortality.[5] Hence, other non-invasive methods are more preferable. Multi slice computed tomographic angiography (MSCTA) of vasculature was first introduced by Rubin et al.[6] Previously, slow image acquisition rate and poor Z-axis resolution to cover whole arterial length (single detector spiral CT) of this modality had limited its reliability.[7][8][9] Today due to its perfect visualization of the entire arterial length and cut-off and run-off sites, special tendency exists toward its use for the evaluation of peripheral vasculature and aorta.[5][10]

There are previous studies in the literature evaluating quality, accuracy and technical aspects of MSCTA for the diagnosis of peripheral vascular disease (PVD) and the aorta,[11] some of which have compared and mentioned its advantages over conventional digital subtraction angiography (DSA).[12][13] However, to our knowledge there are no previous studies comparing the accuracy of this new modality with surgical findings.

We aimed to compare the results of MSCTA as the latest imaging modality for vascular mapping of run-off and cut-off sites with surgical findings as a standard of reference to evaluate the feasibility of this new diagnostic modality in radiology, for which the correct diagnosis is the ultimate goal, as a suitable alternative that may add to the diagnostic accuracy of DSA as a gold standard.

## Patients and Methods

### Participants

Thirty eight patients with clinical signs and symptoms suggestive of AD were assessed using 64-MSCTA. All vascular surgery candidate patients who had established indications of MSCTA, including PVD such as critical ischemia and claudication and renal, mesenteric or hepatic vascular occlusive disease were included.[14] The exclusion criteria were renal insufficiency (serum creatinine >2 mg/dl), contraindication for the use of iodinated contrast medium, respiratory failure and congestive heart failure. Written informed consent was obtained from all participants. Patient information was saved in the hospital data base and only the investigators had access to it.

Diagnosis of AD was established by physical exam such as pulse control, bruit auscultation, measurement of ankle brachial index (ABI), evaluating the color and nutritional status of the skin and comparing the extremities together.[2] CT angiography by 64-MSCTA scanner was performed for all patients to evaluate the diagnosis and conclude the best surgical approach.[15] This was a prospective cross sectional study conducted on 38 patients from March to February, 2009 in Radiology and General Surgery Research Centers of Imam Khomeini hospital of Tehran University of Medical Sciences. The Institutional Review Board (Ethics Committee of Tehran University of Medical Sciences (TUMS)) approved this study.

### Test Methods

We used surgical findings as a standard of reference, because the accurate localization and determination of the severity of arterial involvement is of great significance, and to evaluate the feasibility of this new diagnostic modality in radiology, for which the correct diagnosis is the ultimate goal as a suitable alternative.

One day prior to the operation, a fast speed 64 detector CT scanner (GENERAL ELECTRIC, Milwaukee WI.) allowing continuous acquisition of cranio-caudal direction from the xiphoid process to pedal arteries in about 30 seconds was employed. The slice thickness was 0.625 mm in the upper extremities and 1.25 mm in the lower extremities and the weighted CT dose index was 17 mGr with one second gantry rotation time. The tube current and voltage were 145 mA and 120 kV, respectively. The mean scan time was 0.80 S and the pitch value ranged from 0.6 to 1.0. Patients were laid in the supine position on the CT table with the feet directed towards the gantry and 140 ml of nonionic, contrast medium (Visipaque 320 mg/mL, Amersham Health, Buckinghamshire, UK) was injected into the antecubital vein at a flow rate of 5 mL/s with a power injector, through an 18-22 G inserted cannula. The images were rendered anonymously and transferred in a separate folder on the graphic console independently.

Revascularization and exploration of PVD was performed intraoperatively following MSCTA in order to determine the extent of stenosis. Distal bypass grafting was performed using the reversed saphenous vein or synthetic prothesis graft technique under general anesthesia. Arterial clamping was performed after intravenous injection of unfractionated heparin (5000 IU), and autologous heparinized vein or synthetic prosthesis was transplanted. Surgical findings including cut-off and run-off sites and other relevant pathologies were recorded intraoperatively. Surgical and MSCTA findings were compared, considering the surgical findings as gold standard, appraising the accuracy of this imaging method as an alternative diagnostic modality analyzed by two radiologists with experience in vascular and interventional radiology and CT imaging of vessels and were blinded to the results of the surgery.

### Statistical Methods

Data analysis was carried out using SPSS 16 for Windows (SPSS Inc., Chicago, IL). The sensitivity, specificity, positive predictive value (PPV), negative predictive value (NPV) and the accuracy of MSCTA compared to surgery was calculated. Comparison was performed with the chi-square test to test for any significant difference between MSCTA and surgery regarding the diagnostic value of MSCTA angiography in the diagnosis of PAD compared with surgical findings as a standard of reference. Spearman’s correlation coefficient was used with respect to inter-rater reliability to measure pair wise correlation among raters. [16]

## Results

### Participants 

**Fig. 1:**
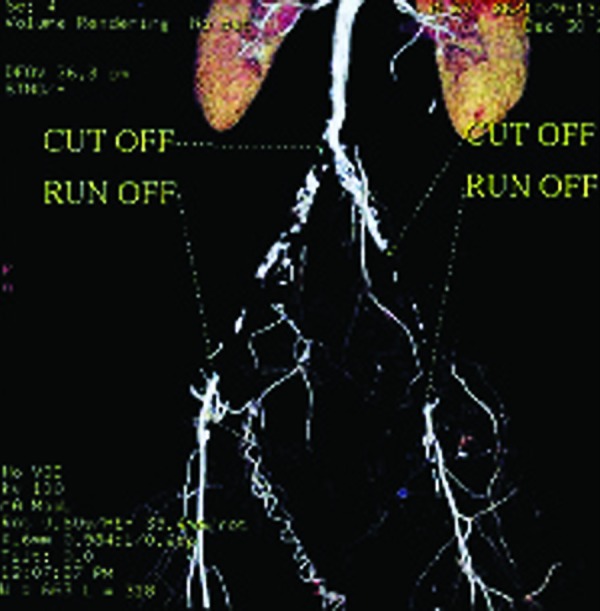
64-MSCTA image in a 67-year-old man. Aorto-iliac and peripheral cut-off and run-off study in a patient with stenosis of aortoiliac bifurcation.

**Fig. 2:**
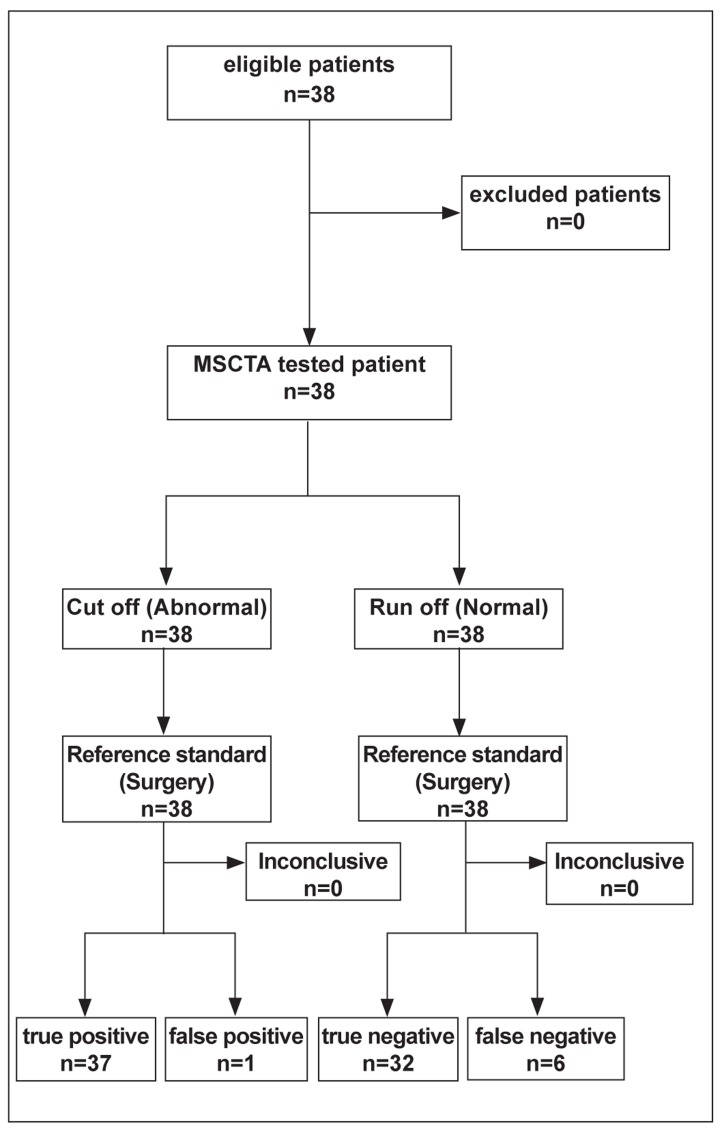
Flow diagram of patients’ recruitment

This study was conducted from March to February, 2009 in Radiology and General Surgery Research Centers of Imam Khomeini Hospital of Tehran University of Medical Sciences. Totally, 38 patients were enrolled in the study with the mean age of 59.34±15.86 (range, 23-93) years, consisting of 26 (68%) males and 12 (32%) females. None of them had a history of revascularization and all of them were visited in the vascular clinic due to an acute or chronic symptomatic vascular event. All these cases had an indication for vascular surgery and all underwent 64- MSCTA (Figs. 1 & 2).

### Test Results

**Fig. 3:**
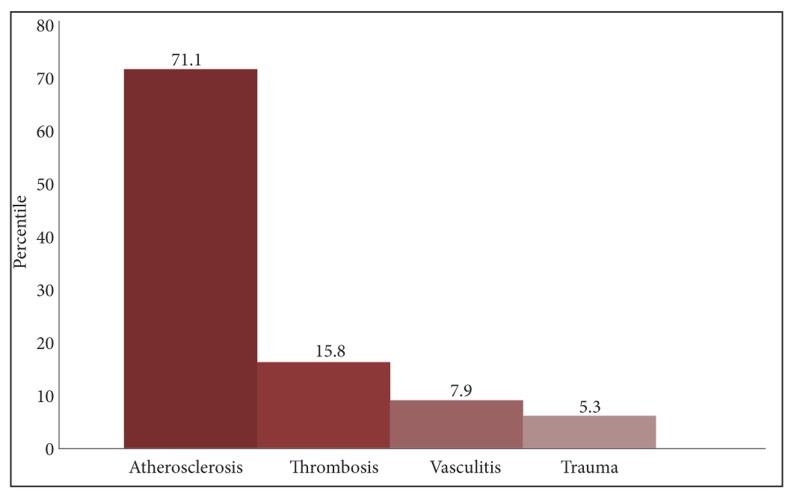
Distribution of etiologic factors of arterial disease.

**Fig. 4:**
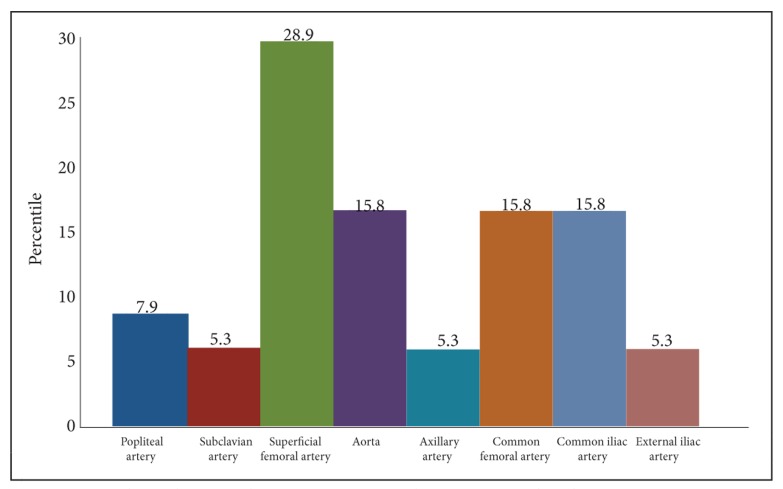
Distribution of the involvement of different arteries.

One day prior to the operation, MSCTA was carried out. The most common causes of vascular occlusion were atherosclerosis in 27 (71.1%) of the patients, thrombosis in six (15.8%) cases, vasculitis in three (7.9%) and trauma in two (5.3%) (Fig. 3). MSCTA results demonstrated the stenosis of superficial femoral artery as the most common site of stenosis in 11 of the patients (28.9%) (Fig. 4). Spearman’s correlation coefficient, measuring the inter-rater reliability presented the value of 0.837 which shows a high degree of agreement between the raters (p=0.0001). Conclusively, AD of the lower extremities 84.2% (n=32) was more common than the upper extremities 15.8% (n=6). There were no adverse events from performing MSCTA.

### Estimates

The true positive, true negative, false positive, false negative, sensitivity, specificity, positive predictive value (PPV), negative predictive value (NPV), accuracy, positive likelihood ratio, negative likelihood ratio and the diagnostic odds ratio of MSCTA compared to surgery is depicted in Table 1. The results of MSCTA were compared with surgery as a standard of reference which showed concordance in the majority of patients equal to 81.6% (n=31). Discordance was observed in 18.4% (n=7) of the results. Cut-off sites were correctly identified by MSCTA in 97.3% (n=37) of the cases. The most common sites of discordance observed in our study were in run-off sites 18.2% (n=6) (Table 2). Additionally, there was no statistically significant correlation between the site (p=0.80), cause (p=0.70) or side (left or right, p=0.97) of stenosis with the concordance of MSCTA and surgical findings.

**Table 1:** Sensitivity, Specificity, Positive Predictive Value (PPV), Negative Predictive Value (NPV), True Positive, True Negative, False Positive, False Negative, Positive Likelihood Ratio, Negative Likelihood Ratio and the Diagnostic Odds Ratio of MSCTA Compared to Surgery

	** Lower Limb**	** Upper Limb**	** Total**	** Confidence Interval 95%**
Sensitivity	83.8%	100%	86%	0.7274 to 0.9344
Specificity	96%	100%	96.9%	0.8468 to 0.9946
Positive Predictive Value	96.8%	100%	97.4%	0.8456 to 0.9986
Negative Predictive Value	81.3%	100%	84.2%	0.6807 to 0.9341
True Positive	31	6	37	
True Negative	26	6	32	
False Positive	1	0	1	
False Negative	6	0	6	
Positive Likelihood Ratio			28.395	4.106 to 196.373
Negative Likelihood Ratio			0.144	0.068 to 0.303
Diagnostic Odds Ratio			197.333	22.548 to 1726.976

**Table 2:** Comparison of the Results of MSCTA with Surgical Findings

		** Concordance**	** Discordance**
Upper Extremities		100% (6)	0% (0)
Lower Extremities		78.1% (25)	21.9% (7)
Total		81.6% (31)	18.4% (7)
Cut-off Sites	Upper Extremities	100% (6)	0% (0)
	Lower Extremities	96.8% (31)	3.2% (1)
	Total	97.3% (37)	2.7%(1)
Run-off Sites	Upper Extremities	100% (6)	0% (0)
	Lower Extremities	81.2 (26)	18.8 (6)
	Total	81.5% (32)	18.2% (6)

**Fig. 5:**
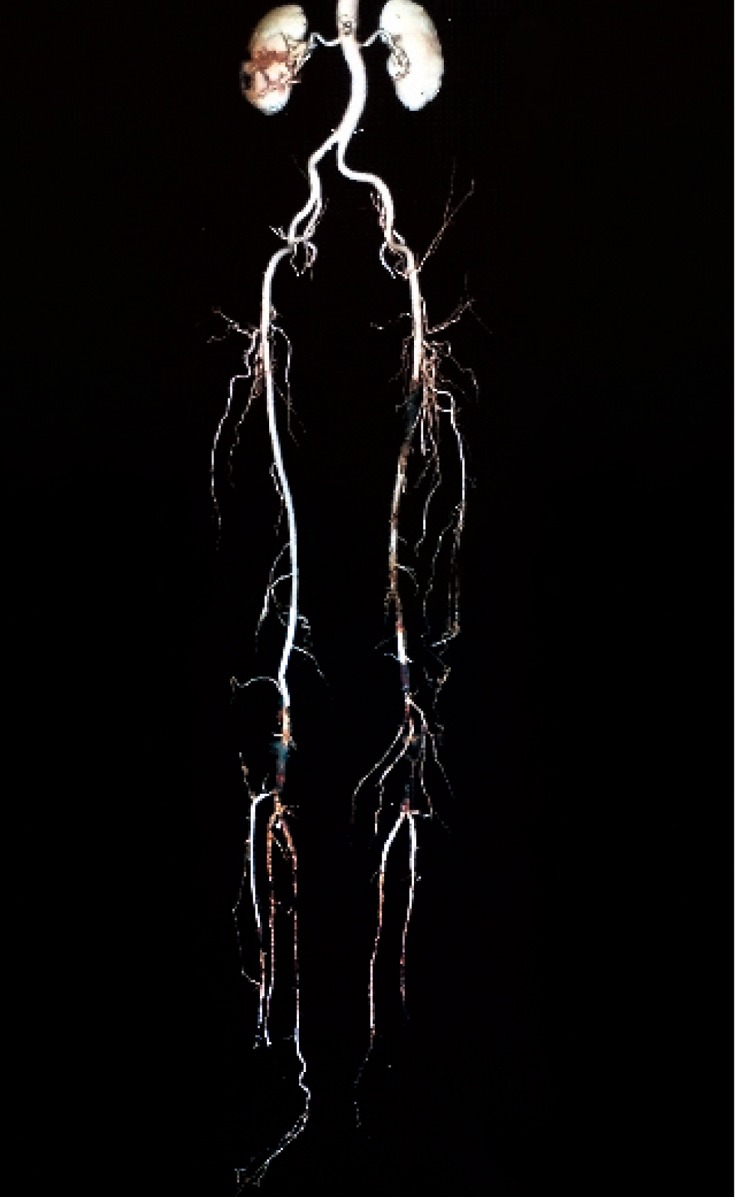
A 75-year-old man with intermittent claudication of the lower limb from 6 years ago, rest pain from 1 year ago and left foot ulcers from 3 months ago. Physical examination showed no palpable popliteal pulses in the lower limbs. MSCTA imaging shows the cut-off point on the proximal part of the superficial femoral artery (SFA) and run-off site in the posterior tibialis and peroneal artery. The discordance was the result of flow overestimation in the left posterior tibialis artery (PTA) in MSCTA image and the degree of mural calcification was more after surgical exploration than that estimated by MSCTA. However, surgical exploration of the posterior tibialis artery demonstrated a very narrow lumen of this artery without considerable flow. The saphenous vein was bypassed from the distal part of the common femoral artery (CFA) to the proximal part of the posterior tibialis artery, but the anastomosis was unsuccessful and the patient’s foot gangrened after seven days.

**Fig. 6:**
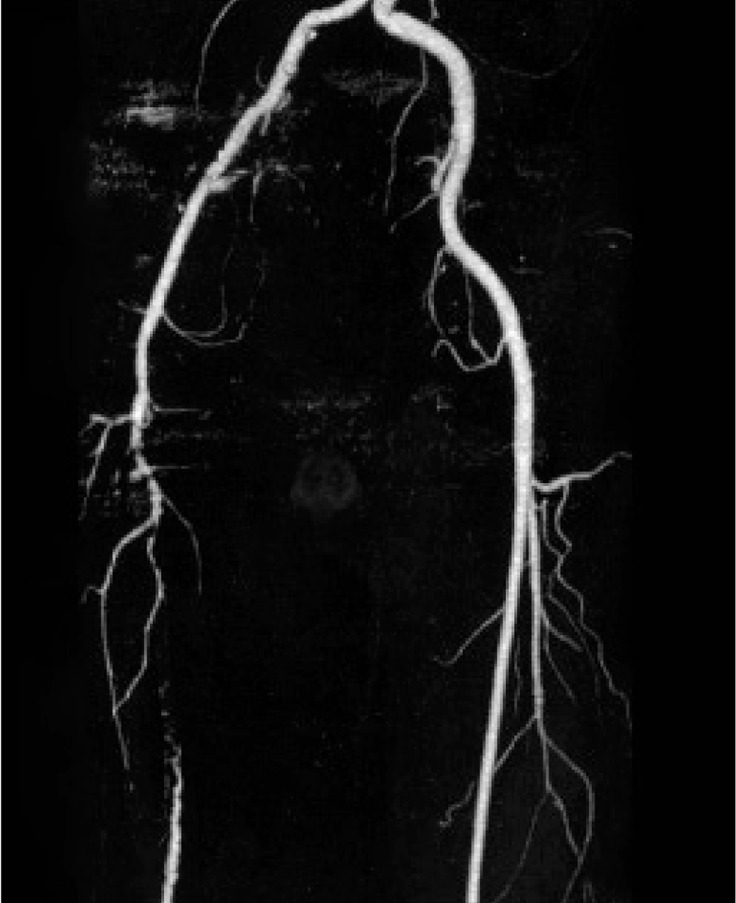
A 58-year-old man with a history of opium addiction. He presented with right foot claudication from last year, right foot rest pain from 6 months ago and ischemic ulcer in the right toe from 40 days ago. Very weak femoral pulses were detected with no pulse in the distal branches. MSCTA showed flow in the middle portion of the superficial femoral artery (SFA), while having thrombotic occlusion in the distal portion of the artery after surgical exploration. The run-off site was seen in the proximal portion of the popliteal artery which was anastomosed to the proximal portion of the right popliteal artery.

**Fig. 7:**
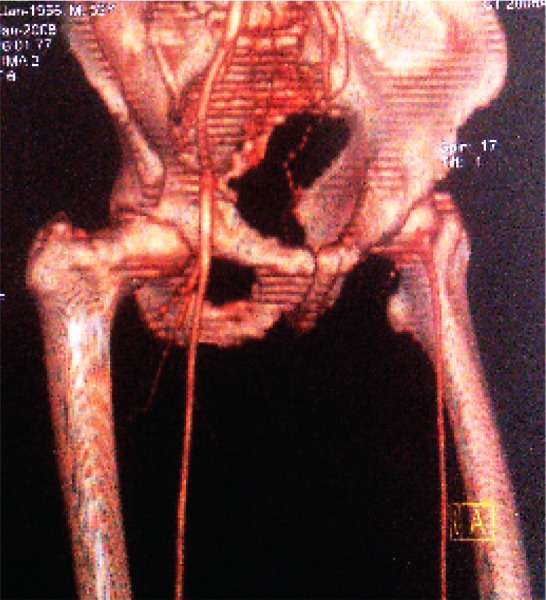
A 50-year-old man, heavy smoker with symptoms of left foot claudication from 3 years ago and rest pain in the left lower limb from 1 month ago. In physical examination, no palpable femoral pulses were detected. In MSCTA, run-off location was distal to the left common femoral artery (CFA), but surgical findings showed thrombosis of the same portion as well as the proximal part of the left SFA. Ten centimeter synthetic bypass graft was grafted to the distal part of the CFA bifurcation site.

**Fig. 8:**
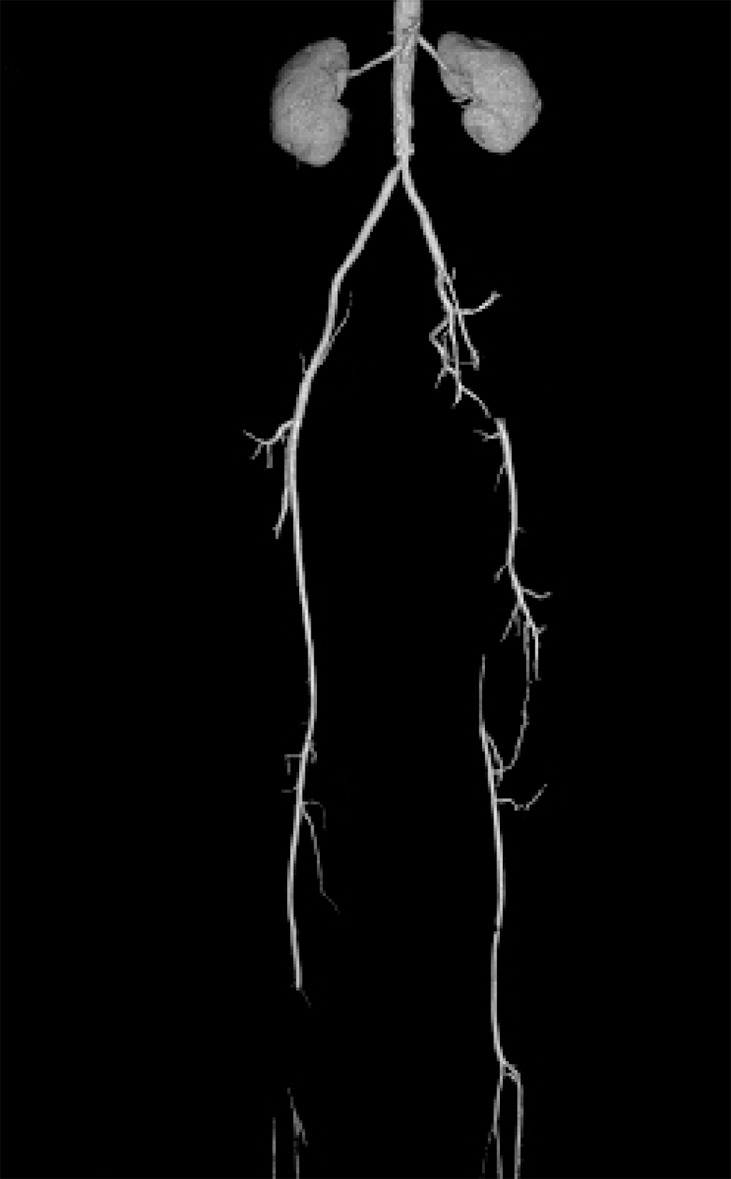
A 38-year-old heavy smoker and opium addict with symptoms of rest pain on the lower limbs, intermittent claudication with signs of severe chronic ischemia of both feet. There were no palpable femoral pulses and atrophy of the left lower limb was considerable. MSCTA shows very good flow on the proximal part of the left profunda femoris and the distal part of the left SFA, but surgical exploration showed flow in the middle portion of the mentioned artery.

**Fig. 9:**
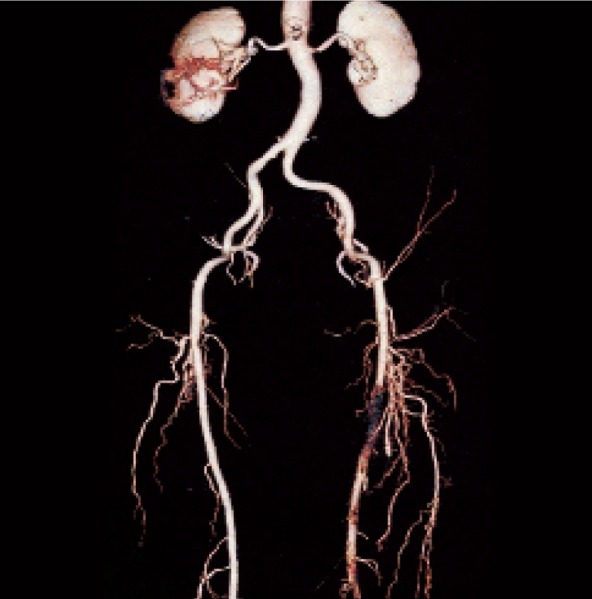
A 44-year-old man, opium addict with symptoms of left foot claudication from last year and left foot rest pain from 8 months ago. Very weak femoral pulses were detected on the left lower limb with no pulses in the distal branches. MSCTA shows very good flow on the proximal part of the left profonda femoris artery (PFA) and the middle part of SFA, but surgery showed no flow in any parts. Additionally, MSCTA shows narrowing in the lumen of the proximal part of the left PFA, which was not detected by surgeons.

**Fig. 10:**
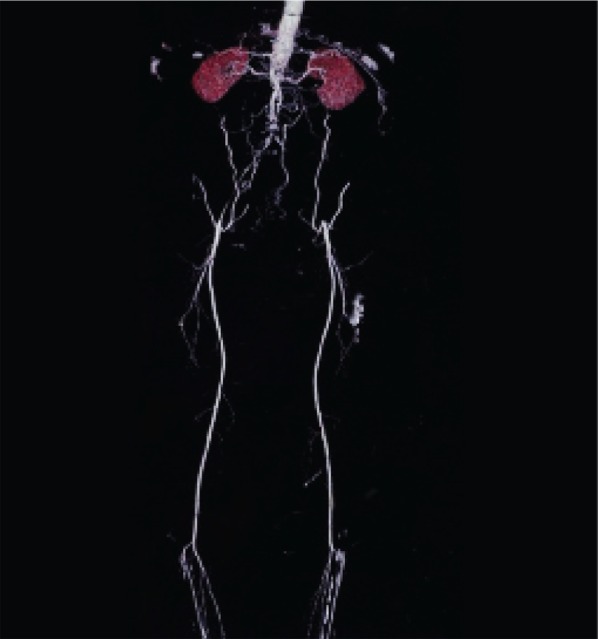
A 62-year-old man with a history of progressive intermittent claudication in the right lower extremity from 5 years ago and decrease in libido. He presented with acute rest pain from 15 hours ago. There were no palpable femoral pulses bilaterally and atrophy of the right lower limb was considerable. In MSCTA, run-off location was distal to the right common femoral artery (CFA), but surgical findings showed thrombosis of the same part and proximal part of the right SFA. Hence, synthetic bypass graft was grafted to ten centimeter to the distal part of the CFA bifurcation site.

**Fig. 11:**
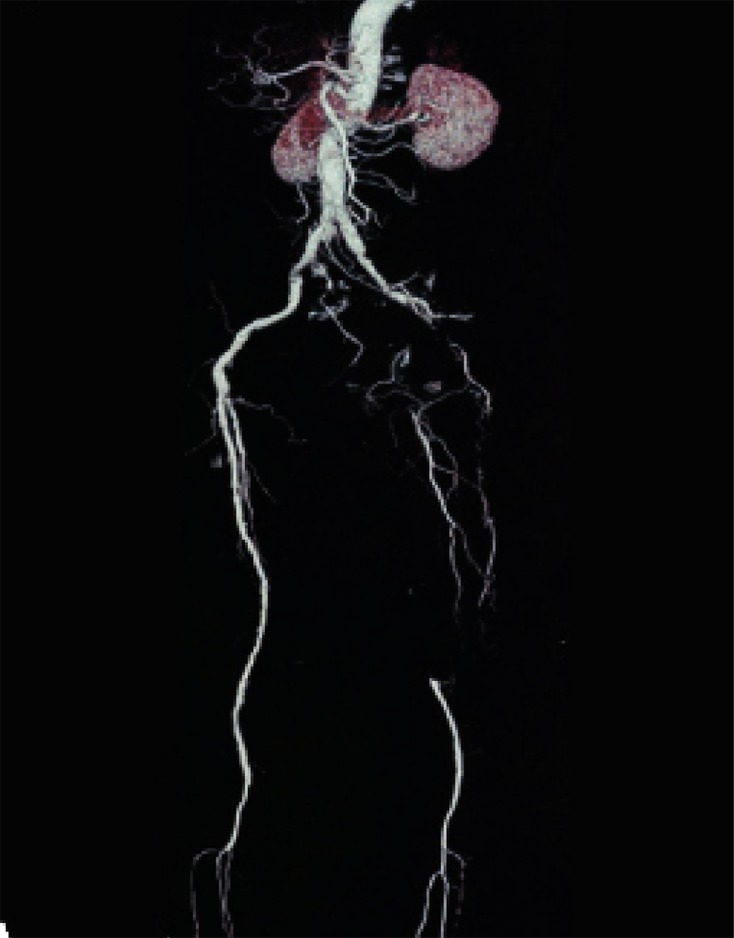
A 74-year-old man, heavy smoker with symptoms of left lower limb claudication for 2 years and ischemic ulcer in the left toe from 2 months ago. We found no palpable femoral pulses on the left lower limb and the left toe was gangrened. MSCTA showed a run-off site of the distal part of the left SFA, while surgical findings showed thrombosis that was anastomosed to the deep femoral artery.

Figs. 5, 6, 7, 8, 9, 10 & 11 illustrate the patients with mismatch findings between MSCTA and surgery.

## Discussion

Arterial disease is a significant cause of morbidity and a poor prognostic factor amongst the elderly [17] and MSCTA as a new diagnostic modality is likely to add to the diagnostic accuracy of CT in vascular disease with enhanced spatial and temporal resolution. Aiming to evaluate the diagnostic accuracy of MSCTA in detecting run-off and cut-off sites, our study showed 81.6% concordance and 18.4% discordance with surgical findings as a standard of reference that was in agreement with result of a previous study [18].

Cut-off sites were correctly identified by MSCTA in 97.3% of cases. Discordance was more prevalent in run-off sites and was detected in seven cases (six cases in run-off and one case in cut-off site) that were probably due to flow overestimation. Moreover a very narrow lumen or thrombosis in CFA, SFA and PTA were found where run-off and cut-off sites were not suitable for anastomosis. Additionally, according to our findings, MSCTA has an admissible sensitivity, specificity, PPV, NPV and accuracy compared to surgery especially in the upper extremities,

The diagnostic approach of patients suspected of AD has changed dramatically during the past decade. Different diagnostic modalities such as color Doppler ultrasonography, angiography, CT angiography and magnetic resonance angiography are used for this purpose. The gold standard and diagnostic test of choice for AD is angiography although its invasiveness, mortality, morbidity and complications risk (1%) besides the significant improvement in sensitivity and quality of other noninvasive modalities has limited its use [19]. DSA has many complications such as catheter related and puncture site complications that are directly due to angiographic procedures [20].

Many studies acclaim MSCTA can reduce the total contrast agent injection and hypersensitivity reactions to contrast media [1]. MSCTA has two distinct advantages over DSA in detection of AD. Firstly it can accurately demonstrate the intra arterial occlusive sites, degree and recanalization of thrombosis by multi planar cross sectional reformatted MSCTA, whereas additional lateral and oblique views are necessary for DSA [12]. Secondly, MSCTA is capable of demonstrating the distal portion of stenotic segment which could not be opacified by DSA [13].

Various vascular studies on CT angiography of aortic dissection and aneurysm, renal and carotid occlusion and stenosis of aortoiliac and extremities vascular bed have been published since 1991. Lawrence et al. compared CT angiography and DSA of lower extremities where both cut-off and run-off sites showed good correlation [7]. They employed two sequential helical CT angiography of aortoiliac segment like two other studies using CT angiography for the evaluation of vascular tree in trauma [21][22] but all of them studied only a segmental region, whilst we assessed the entire vascular tree with 64-MSCTA. Multidetector CT angiography was first used by Rubin et al. [6] in 2001 and the results of other studies comparing MSCTA and DSA introduced MSCTA as an accurate and reliable noninvasive alternative method in assessment of AD either [19].

Other inexpensive, noninvasive methods such as Doppler ultrasonography can also be used for this purpose but the less popularity of this method is due to its shortcomings that are being operator dependent [23]. Magnetic resonance angiography (MRA) with gadolinium contrast has also a good accuracy in evaluation of lower limb vasculature although it has significant artifacts in intra mural arterial calcifications and is contraindicated in patients with metal stents or pace makers [24].

A limitation of our study is that one of the major concerns of MSCTA is the dose of ionizing radiation delivered to the patient. As we mentioned previously the weighted CT dose index was 17 mGr which is high and might influence our usage. As we know, the optimization of radiation dose during MSCTA has been the topic of many studies [25][26][27][28]. Hence, further studies with larger sample sizes and more powerful statistical analysis is recommended to deal with this shortcoming. Moreover the nephrotoxicity of non ionic contrast agents has been significantly reduced compared with that of ionic contrast agents and the administration of these non ionic agents can thus be considered safe and well tolerated, even in high risk populations [29]. The goal of this study was to validate the possibility and feasibility of performing routine MSCTA angiography, which could be a suitable alternative and may be a viable choice, for routine clinical diagnosis.

In conclusion, MSCTA findings were compared with surgery as a standard of reference and showed concordance in the majority of cases, hence this novel diagnostic modality, could be a suitable alternative and may be a viable choice, for routine clinical diagnosis.
